# Plant Tolerance to Drought Stress with Emphasis on Wheat

**DOI:** 10.3390/plants12112170

**Published:** 2023-05-30

**Authors:** Sarah Adel, Nicolas Carels

**Affiliations:** 1 Genetic Department, Faculty of Agriculture, Ain Shams University, Cairo 11241, Egypt; sarah_adel@agr.asu.edu.eg; 2 Laboratory of Biological System Modeling, Center of Technological Development for Health (CDTS), Oswaldo Cruz Foundation (Fiocruz), Rio de Janeiro 21040-361, Brazil

**Keywords:** ChIP, climate change, epigenetic, genomics, histone code, QTL, transcription factors, transgenic crops

## Abstract

Environmental stresses, such as drought, have negative effects on crop yield. Drought is a stress whose impact tends to increase in some critical regions. However, the worldwide population is continuously increasing and climate change may affect its food supply in the upcoming years. Therefore, there is an ongoing effort to understand the molecular processes that may contribute to improving drought tolerance of strategic crops. These investigations should contribute to delivering drought-tolerant cultivars by selective breeding. For this reason, it is worthwhile to review regularly the literature concerning the molecular mechanisms and technologies that could facilitate gene pyramiding for drought tolerance. This review summarizes achievements obtained using QTL mapping, genomics, synteny, epigenetics, and transgenics for the selective breeding of drought-tolerant wheat cultivars. Synthetic apomixis combined with the msh1 mutation opens the way to induce and stabilize epigenomes in crops, which offers the potential of accelerating selective breeding for drought tolerance in arid and semi-arid regions.

## 1. Introduction

Agriculture is an essential economic activity, as well as a source of food and jobs, which makes its sustainability mandatory [1]. However, the quantity and quality of cultivated crops depend on local weather variables [2] and particularly on water availability. The global warming that we now observe [3] makes it difficult to predict the type of challenges that selective breeders will face to guarantee food security in the near future [4]. Nonetheless, selective breeding for increased tolerance to drought is vital [5] to mitigate inevitable pressures on water supply [6]. With the deficit of water progressively increasing [7,8,9], its value is being assessed more carefully [10]. In any case, the consequences of global warming suggest that societies will have to adapt their behavior to reach sustainability [11] since water shortages and/or unpredictable supply from one year to the next will remain a recurrent problem for farmers from now on [12,13].

In the mid-XX century, agriculture changed the course of its evolution through the so-called Green Revolution, i.e., the introduction of cultivation protocols involving high-yielding varieties, chemical fertilizers, pesticides, irrigation, and mechanization. Today, together with maize, rice, and soybean, wheat represents a staple food for humanity [14]. However, the pressure for the continuous increase of food production by agriculture is becoming critical, despite the necessity of biodiversity preservation and climate change mitigation. Because wheat is a primary source of calories and proteins, biotic and abiotic stresses may constitute bottlenecks for the human population, whose size continues to grow. In particular, rust disease, a fungal pathogen of cereals, is seen as a foremost threat to the sustainable growth of wheat production [15,16]. Indeed, agricultural practices will need to keep pace with the intensification of sustainable food production to face the challenge of feeding a world population estimated to reach over nine billion by 2050 [17,18].

Since drought is a major hurdle that limits crop yields, further investigations of wheat drought tolerance-related processes are critical for the genetic improvement of drought tolerance in this crop [19,20].

A document from the Intergovernmental Panel on Climate Change [21] indicated that dangers emerging from climate change could worsen the risks, vulnerability, and unpredictability facing humans and ecosystems. To address these threats, the European Union issued policies in 2021 to “*adapt to climate change in a consistent manner in all policy areas*” and to “focus, in particular, on the most vulnerable and impacted populations and sectors” [22]. Taking Egypt as an example, people rely exclusively on irrigation to produce about 9 million of the 20 million tons of wheat consumed annually and import the other half. Because Egypt is currently the largest wheat-importing country in the world, it tries to become less reliant on imports by increasing its production [23]. The authorities promote the increase of wheat production via (i) vertical expansion by way of offering the wheat farmers all their needs including fertilizers, pesticides, advice, laboratory support, and first-class cultivars, and (ii) horizontal expansion by cultivating wheat in new and reclaimed lands. Hopefully, given its political effort, Egypt may have witnessed in 2019 a ranking of 5th among the regions of high grain productivity [24] with the benefit of introducing new cultivars [25]. Because rain precipitation may decrease in the future, the wheat yield of this country could fall by as much as 9% in 2030 and by 20% in 2060 [3,26].

Because we are concerned about plant adaptation for tolerance to drought-stress, with particular emphasis on wheat production challenges in the era of climate change, we focused this review on selective breeding through genetics and epigenetics in an effort to integrate data and concepts. We concluded that synthetic apomixis combined with the msh1 mutation opens the way to induce and stabilize epigenomes in crops, which offers the potential for accelerating selective breeding of drought-tolerant crops for arid and semi-arid regions.

## 2. QTL Mapping for Drought Stress Tolerance in Plants

Drought-tolerance refers to polygenic traits or quantitative trait loci (QTL) identified in crop plants (Table S1a,b). Conceptually, drought tolerance occurred during the transition of plants from water to land, and the role of drought tolerance was to allow this transition [27]. Some of the drought-related-QTLs are associated with root architecture, plant biomass, water soluble carbohydrates, membrane stability index, and grain yield. Some of these QTLs are linked to agronomic or physiological traits associated with drought-stress and can contribute up to 20% phenotypic variation [28]. Examples of QTLs involved in drought stress tolerance and their related candidate genes in model plants are given in Table S2 [29]. For some other interesting references on QTLs of drought tolerance in crops of economical importance, see [30,31,32,33,34,35,36,37,38,39,40,41,42,43,44,45,46,47,48].

Genome-wide association study (GWAS) of wheat has been used to map QTLs associated with seedling drought tolerance [49]. Multiple significant QTLs associated with seedling drought tolerance were identified on chromosomes 1B, 2A, 2B, 2D, 3A, 3B, 3D, 4B, 5A, 5B, 6B, and 7B. Twelve stable QTLs responded to drought stress for various traits; among them were shoot length and leaf chlorophyll fluorescence, which were good indicators of drought stress tolerance. Some QTLs of wheat detected by Maulana et al. [49] co-localized with previously reported QTLs (i) for root and shoot traits at the seedling stage and (ii) for canopy temperature at the grain-filling stage. Some significant single-nucleotide polymorphisms (SNPs) were identified in candidate genes involved in plant abiotic stress responses, which will allow marker-assisted selective breeding for drought tolerance at the seedling stage [50].

**Figure 1:**
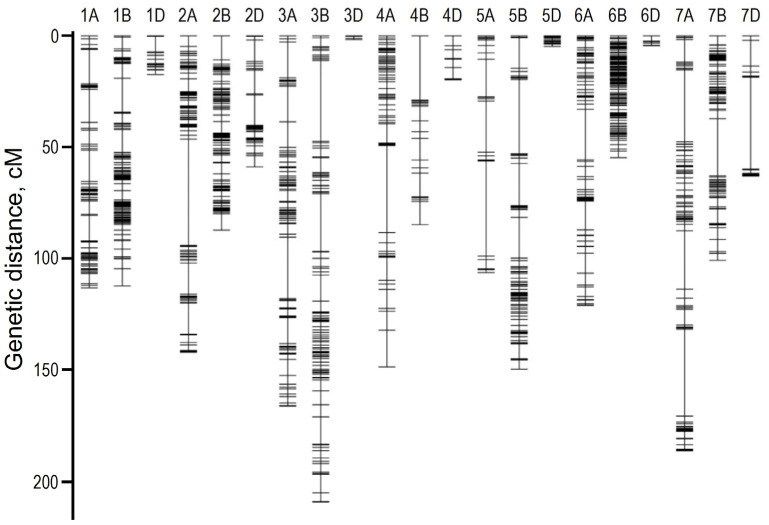
Linkage map constructed from genotyping-by-sequencing derived SNPs in a recombinant inbred population obtained from a cross between drought-tolerant (Harry) and drought-susceptible (Wesley) lines of wheat (adapted with permission from ref. [53], Copyright 2017 owned by Nature. More details on “Copyright and Licensing” are available via: https://creativecommons.org/licenses/by/4.0/).

The combination of GWAS and QTL mapping at seedling stage has revealed candidate genes and SNP networks controlling traits of recovery and tolerance associated with drought in seedlings of winter wheat [51]. Mathew et al. [52] found that drought tolerance and drought-recovery were only weakly correlated. Interestingly, most QTLs associated with drought-recovery were different from those associated with drought tolerance. These authors also colocalized SNPs with 14 genes, as shown in Table S1a. The markers complete the genetic map of QTLs for drought tolerance that was developed by Hussain et al. [53] by mapping SNPs obtained through crossing drought-tolerant (Harry) and drought-susceptible (Wesley) lines (Figure 1).

**Figure 2:**
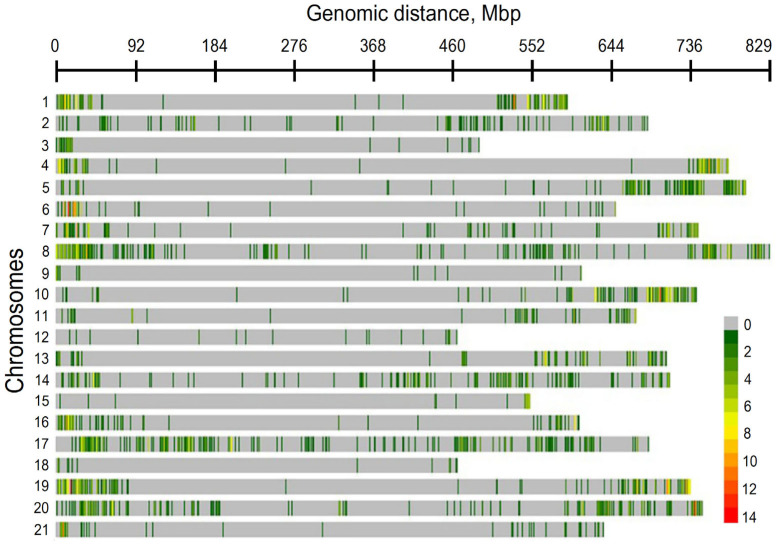
Distribution of SNPs on wheat chromosomes in a biparental mapping population derived from crossing drought-tolerant (Harry) and drought-susceptible (Wesley) inbred lines (adapted with permission from ref. [51], Copyright 2022 owned by Elsevier. More details on “Copyright and Licensing” are available via: https://creativecommons.org/licenses/by/4.0/).

The genetic map by Hussain et al. [53] clearly shows a distortion of the physical map of drought-associated SNPs as reported by Sallam et al. [51] (Figure 2). The biased distribution of transcriptionally active regions along cereal chromosomes was also reported by other authors [54,55]. The same observation was made for QTLs associated with other traits [48]. Despite regular Giemsa banding along *Hordeum vulgare* [56], Figure 2 clearly shows that pericentromeric areas are generally empty of drought-associated SNPs, which tend to be more frequent at the distal chromosome extremities. This trend has been recognized since the decade of the 1990s [57,58,59,60] and suggests a distal preference for transcriptionally active genes along plant chromosomes [61] correlating with a higher crossing-over frequency [60,62].

The high rate of synteny in Poaceae [63,64,65,66] is expected to help to find orthologous genes in other cereals [67]. Thus, it is interesting to look at genes for drought tolerance in other plant crops of this group and even in other plant models [48,68,69,70,71,72]. In this sense, a methodology was developed to identify key drought-adaptive genes and the mechanisms in which they are involved, to test their evolutionary conservation. Empirically defined filtering criteria were used to facilitate a robust integration of microarray experiments from Arabidopsis, rice, wheat, and barley available in public databases [73].

Sorghum (*Sorghum bicolor* L. Moench) is a C4 cereal crop adapted to different environmental conditions, including drought-prone areas where many other staple crops fail [74]. It is among the most drought-adapted cereal crops, but its adaptation is not yet well understood. Transcriptome analysis of drought tolerance at the seedling stage revealed that about half (n = 70) of the up-regulated genes in response to drought were novel with no known function and the remainder were TFs, signaling and stress-related proteins implicated in drought tolerance in other crops [75].

In sorghum, premature leaf death occurs when water starts to be limiting during the grain filling stage. Premature leaf senescence, in turn, leads to charcoal rot, stalk lodging, and significant yield loss. Most sorghum cultivars have pre-flowering drought tolerance, but many do not have any significant post-flowering drought tolerance. Stay-green is one form of drought-tolerance mechanism giving sorghum a tolerance to premature senescence under soil drought-stress during the post-flowering stage. The stay-green trait results in larger functional photosynthetic leaf area during grain filling and even after physiological maturity [76]. In sorghum, four genomic regions associated with the stay-green trait were identified in all field trials and accounted for 53.5% of the phenotypic variance [77]. In wheat, deciphering stay-green revealed variations in the exons of *CaO* and *RCCR* associated with a significant difference in the regulation of *CaO* and *Cab* at seven days after anthesis under terminal heat stress [78].

## 3. Genomics

As outlined above, plant species may use various drought adaptive mechanisms depending on their biological setup, which reflects an adaptive evolutionary genome strategy, given their environment. Still, for most plant species, the eco-physiological and genetic mechanisms underlying variation for drought adaptation in the field are not known. Thus, deciphering the mechanisms of drought adaptation remains a foremost challenge in plant biology and breeding [79,80,81].

### 3.1. Genome Phenotype and Genome Strategy

In vertebrates, the genome was shown to be organized into two major components with specific functionalities [82] according to guanine plus cytosine (GC) content. The base changes during vertebrate evolution were such that the GC-poor compartment remained poor in cold-blooded vertebrates (fishes, amphibians, reptiles), but their GC-rich compartment became even richer in GC during their transition to warm-blooded vertebrates (mammals and birds). This compositional transition led to a range of functional adaptations at the molecular level [82]. The effect of this major transition [83] on the genome structure of modern species of both groups can still be observed through GC composition at the regional level, which led Bernardi to coin the term isochores in 2007 for DNA stretches larger than 300 Kbp that do not vary by more than an average standard deviation of ~2% GC [84].

The concept of genome phenotype correlates with that of genome strategy and this last concept was recognized through codon usage analyses by Grantham et al. [85] and extended to plant genomes by Carels [86]. Indeed, as noted by Grantham et al. [85] “Systematic third base choices can be used to establish a kind of genetic distance, which reflects differences in coding strategy. The patterns of codon choice we find seem compatible with the idea that the genome and not the individual gene is the unit of selection. Each gene in a genome tends to conform to its species’ usage of the codon catalog”. By extension, Grantham et al. [85] suggested that selection acts on genomes by shaping coding sequences through their codon usage, which would comprise an adaptive strategy given an evolutionary history resulting in particular features of the genome phenotype. Carels [86] recognized the difference in the organization of intergenic sequences in humans and maize despite their similarity in genome size and bias of GC composition of codons. This is particularly clear when comparing the compartmentalization in the maize gene space to the compartmentalization in the human genome, which shows that the compositional regression line of GC in the 3rd position of codons with intergenic sequences is too steep in maize to have a complete separation between GC-poor and GC-rich compartments. The slope of the maize orthogonal regression line is about four times steeper than that of the human one, which leads to about seven times the contraction of the gene space compared to humans. As a result, only ~3% GC separates the GC-poor and GC-rich compartments. Interestingly, zein genes, which are expressed only during the embryonic phase, are located in the GC-poor compartment [86]. This situation mimics the expression of embryonic genes in the vertebrate GC-poor compartment. More recently, the concept of genome strategy as a response to adaptation to environmental challenges was recognized in bacteria [87]. Surprisingly, almost all the interval of GC variation observed among bacterial species [88], the so-called universal correlation, is found within the genomes of species of warm-blooded vertebrates. This implies that genes are spread over GC-poor as well as GC-rich contexts offering a richer range of promoter sequences for interaction with TFs than might be the case with only one GC-poor compartment. Therefore, one may see in genome compartmentalization according to regional composition an opportunity for a more diverse genetic code exploration on which environmental selection may operate. A type of driving adaptation that has been proposed is the maximization of energy use in challenging conditions [89]. Another functional correlation that could also operate is a trend for larger use of beta-sheets in proteins from GC-poor genes and of turns in proteins from GC-rich genes [89].

Considering plants, it is interesting to note here that a compositional transition occurred in Poaceae compared to other monocots and dicots [90], which is very clear when considering the third positions of codons [91,92]. However, when considering the intergenic sequences, the transition occurred in the shifting mode [93] and an isochore structure typical of warm-blooded vertebrates could not be found [86]. By contrast, the existence of the GC-rich compartment inside the gene space of maize indicates that some long-term process of regional GC enrichment mechanism has played a role in the genome compartmentalization at sequences of a size around ~100 kb, in the past history of maize. This compartmentalization that results from the correlation between the compositional distribution of GC-poor, GC-rich genes, and ~100 kb sequences is striking since it fits with the compositional distribution and interspersion rate of retrotranspon families in the gene space. The organization in GC-poor and GC-rich compartments still exists with the gene space preferentially within the GC-rich compartment [86]. The absence of an isochore structure may reflect the different chromosome and nucleus organization between plants and vertebrates.

Vertebrates have a complex Giemsa banding that correlates with isochore distribution [94,95] and nucleus spatial organization [95,96] following the fractal globule model of chromosome distribution [97,98]. The degree of DNA condensation of human chromosomes is relatively low compared to that of plants for which there may be more associated proteins on their surface; hence a longer digestion time is needed [56,99] for karyotype preparation. Since transcriptionally active regions show a distal preference for gene location along plant chromosomes (especially in the cases of large genomes) as in vertebrates [54,55,61,84,100], one may conclude that pericentromeric euchromatin islands are richer in GC-poor genes [101,102], which are known to be less active transcriptionally [61,103] and under other regulation processes [104].

In contrast to warm-blooded vertebrates where centromeric regions and heterochromatine are packed on the nucleus membrane, while euchromatin loops toward its center [95], interphase plant cells present two types of spatial organization [105]: (i) Rosette, in Arabidopsis, i.e., heavily methylated interspersed heterochromatin segments of chromosomes that interact with their centromers (B compartment) to form chromocenters, while euchromatin (A compartment) oozes from chromocenters as loops spanning 0.2–2 Mbp. Euchromatin loops are rich in acetylated histones, whereas chromocenters contain less acetylated histones [106]. (ii) Rabbi, in cereals, i.e., telomeres and centromeres of chromosomes cluster at two different poles in the nucleus [107]. The interphase chromosome distribution in Arabidopsis follows the fractal globule distribution as in vertebrates [108], but the 3D distribution of Rabbi chromosomes is quite different since they have a parallel spatial arrangement [109]. These different chromosome distributions in interphase cells of vertebrates and plants are another layer of genome phenotype corresponding to a genome strategy coding for a molecular strategy pervading the whole cell machinery [89,91,110]. Such a biological framework cannot be modified within a short time, which means that it can be, eventually, negatively selected and lead to extinction in case of low fitness for drastic environmental changes since it implies a long evolutionary history correlated to evolutionary inertia to produce them.

It is clear from the discussion above that Poaceae occupy a special place in the spectrum of genome organization in angiosperms. Even if intergenic sequences are relatively homogeneous in composition, GC-rich genes have CpG islands in their promoter regions, while this is not the case for GC-poor genes [93]. Since recombination appears to be redirected to gene promoters and CpG islands [111] in humans, it is likely to be the case in plants as well because of the similarity of chromatin features between both biological systems. As outlined above, these regulatory domains may indicate adaptative peculiarities in Poaceae compared to other dicots and monocots, such as a potential for higher tolerance to various abiotic stresses.

### 3.2. Wheat Genome

During GFAR [112], it was stated that scientists from the International Maize and Wheat Improvement Center (CIMMYT) would sequence the genomes of 15 wheat varieties from breeding programs worldwide. This initiative enabled scientists and breeders to identify much faster the key genes able to improve yield as well as tolerance to heat, drought, pest, and other important crop traits.

Wheat is the largest crop genome decoded to date [113]. For sake of comparison, the largest plant genome to date is the Japanese andromeda (*Paris japonica* (Franch. and Sav.) Franch.) with 149 Gbp and the smallest is *Arabidopsis thaliana* L. with 125 Mbp. The wheat haploid genome (~17 Gbp) is about five to six times larger than the human one, so it is a complex structure comprised of three independent genomes. Each sub-genome accounts for ~5.5 Gbp [114]. The high proportion of repetitive DNA (retrotransposons covering >80% of the genome) [115] makes it difficult to decrypt the genome structure of this crop. However, the mapping of markers such as (i) expressed sequence tags (EST), (ii) SNPs, and (iii) contigs (see: https://wheat.pw.usda.gov/cgi-bin/GG3/browse.cgi, accessed on 14 January 2023) is helping in this task and a complete genome sequence is being regularly updated by Ensembl (see: https://plants.ensembl.org/Triticum_aestivum/Info/Index, accessed on 14 January 2023). Until now, more than 1.07 million wheat ESTs were sequenced [116,117], and there are BAC libraries for each of the three sub-genomes (A, B, and D) covering each specific chromosome or chromosome arm. According to the IWGSC Annual Report from 2021, the “WGSC Annotation v2.1 contains 266,753 genes, comprising 106,913 high confidence (HC) genes and 159,840 low confidence (LC) genes” (https://www.wheatgenome.org/content/download/4298/40943/version/1/file/2021_IWGSC_AnnualReport.pdf, accessed on 14 January 2023). BLAST searchable wheat genomic sequences are now available at https://wheat.pw.usda.gov/cgi-bin/seqserve/blast_wheat.cgi (accessed on 14 January 2023).

When investigating the genome of *T. aestivum* cv. ‘Chinese Spring’, *T. urartu*, *Aegilops speltoides*, *T. turgidum* cv. Cappelli, *T. turgidum* cv. Strongfield by whole genome high-throughput shotgun sequencing, class I retroelements were confirmed to be the most abundant. Compared to B, class II mobile elements (DNA transposons) were more frequent in the A and D genomes, respectively [115].

The diploid wheat A and D genome were sequenced first to detect their homologous genes. Shotgun sequencing of wheat genome was initially performed with SOLiD, 454, and Illumina [118]. However, the large quantity of repeat DNA made it difficult to assemble the complete genome. To overcome this difficulty, sequencing was combined with SNP markers, physical and genetic mapping [119,120,121]. GWAS combined with Targeted Induced Local Lesions in Genome (TILLING) was used to annotate the tetraploid and hexaploid wheat gene function as well as to characterize the genetic diversity and ancestral relations between populations [122]. In drought conditions, a larger number of genes differentially expressed were mapped on the B genome than on the other subgenomes, particularly on chromosomes 3B, 5B and 2B. These genes were involved in 116 different pathways [123].

### 3.3. Genomic Contribution to Understanding the Molecular Bases of Wheat Response to Stress

Plants offer a living context for a wide range of molecular interactions to occur among different stress factors. The large number of genes controlling abiotic stress [124] is a challenge that obliged the adoption of multi-omics strategies [125].

Since map-based cloning of genes for tolerance to abiotic stresses is still difficult with forward genetic strategies in wheat (by seeking the genetic basis of a phenotype—typically by inducing mutations), reverse genetic methods (by seeking to learn which phenotypes are controlled by particular genetic sequences) have been widely used to identify heat-responsive genes in wheat. Transcriptome analysis including microarray and RNA-seq are high-throughput strategies to detect differentially expressed genes that were used to analyze the response to heat stress [126]. For instance, differentially expressed genes (DEGs) in wheat grain and flag leaf enabled the identification of three genes, *TaFBR1*, *TaFBR2*, and *TaFBR3*, which may be crucial for wheat’s ability to increase its thermotolerance [127]. The resistance of genes to harmful environmental conditions that could be involved in flavonoid synthesis has been extensively investigated [128]. For instance, a prior study showed that *TaFSL1* improved transgenic Arabidopsis’ ability to tolerate salinity by causing primary root extension when compared to control plants [129]. In another transcriptome investigation, *TabZIP60*, a transcription factor encoding gene, was up-regulated as a result of heat stress and controlled the expression patterns of 1104 genes [130].

Because of their sessile standing, plants are continuously challenged by various biotic and abiotic stresses during their life cycle [131]. To cope with this challenge, plants have evolved complex molecular mechanisms to adapt to these abiotic stresses. As has been shown, DNA methylation and non-coding RNAs (ncRNA) are involved in the post-transcriptional regulation of wheat response to heat [130]. The discovery of miRNAs and their regulatory mechanisms for controlling genes involved in various developmental, biological, and stress responses has advanced our understanding of gene regulation in plants [132]. miRNA appears to target many genes. For instance, in the case of the tolerance to salinity in roots, 75 miRNAs were identified by in silico investigation to possibly target 861 mRNA. The genetic development of wheat cultivars may be aided by this information, which might promote our understanding of how miRNAs mediate the molecular mechanisms of tolerance to abiotic stresses [133].

LncRNAs are a diversified class of RNAs generally defined as transcripts larger than 200 nucleotides that are not translated into protein. LncRNAs include intergenic lincRNAs, intronic ncRNAs as well as sense and antisense lncRNAs involved in the control of transcriptional regulation and genome imprinting in processes such as plant development, disease resistance and nutrient acquisition, through chromatin remodeling, histone modification, pri-mRNA alternative splicing, or acting as ‘target mimicry’ (see refs in [131]). The analysis of differentially expressed lncRNAs, miRNAs, and genes has revealed regulatory networks of lncRNA-miRNA-mRNA modules involved in response to drought stress in wheat [134].

## 4. Epigenetic Modifications

There is growing evidence indicating that plants implement sophisticated epigenetic mechanisms to fine-tune their responses to environmental stresses. Epigenetic processes involving DNA methylation, histone modification, chromatin remodeling, and ncRNAs make up wheat’s response to drought stress. Changes in chromatin, histone, and DNA mainly serve the purpose of memorizing past stress events and enable plants to deal with climate challenges and thrive in stressful environments.

Epigenetics is the study of heritable phenotypic changes that do not involve alteration of the genetic code itself. Epigenetics includes DNA methylation [135], histone modifications, histone variants, and ncRNA modifications that influence the structure and accessibility of chromatin [136] to transcription machinery and alter gene expression [137]. An increased number of investigations have shown the participation of epigenetic mechanisms in the response of plants to abiotic stresses [138]. Therefore, deciphering the epigenetic codes of plant stress response could be of great significance for the selective breeding of drought-tolerant crops [139].

Plants respond through various short-term and long-term strategies depending on whether the stress is permanent or transitory. Short-term strategies include alteration in plant homeostasis while long-term strategies include trans-generational changes involving the development of heritable gene expression changes. This mechanism consists of creating new epigenetic marks while erasing old ones as well as increasing the expression of some genes while silencing the expression of others [140]. Epigenetic reprogramming in response to various environmental challenges contributes to phenotypic diversity as well as tolerance to these challenges [141]. According to Priyanka et al. [141], the mechanism of an epigenetic process can be divided into three stages: (i) Epigenator, which is a trigger, such as foods, toxins, radiation, or hormones, that alters the cells’ environment to produce an epigenetic phenotype. These triggering cues are transient but last long enough to initiate the epigenetic process. (ii) Epigenetic initiator, which translates the epigenator signal into an epigenetic modification of chromatin. An epigenetic initiator is primed by the epigenator and determines the location on a chromosome that should be marked. (iii) Epigenetic maintainer, which sustains the chromatin environment in the current and succeeding generations. Persistence of the chromatin configuration may require cooperation between the initiator and maintainer.

### 4.1. DNA Methylation

As proven by the analysis of Methylation Sensitive Amplification Polymorphism (MSAP), plants can remember their past environmental experience and retrieve their associated information [142,143]. DNA demethylation is an active DNA repair-related mechanism. For example, the Decreased DNA Methylation 1 (DDM1), a nucleosome remodeler involved in the maintenance of DNA methylation, and the Repressor of Transcriptional Silencing 1 (ROS1), a primary factor required for active DNA demethylation, were determined to be involved in UV-B DNA damage repair. A short-term memory induced by a single stress on the parent resulted in epigenetic modifications that were also observed in their first offspring and were reprogrammed in further generations. By contrast, multiple stresses led to long-term memory, and parent modifications were transferred to several generations [144]. Short-term memory allows plants to remain tolerant to certain stresses for up to about 10 days. By contrast, long-term stress memory is regulated by epigenetic modifications and can potentially last for the whole life of plants suffering from continuous stress; ultimately, it may be transferred to the offspring. The transgenerational epigenetic memory of meiotic prophase chromatin features may be inherited from interphase somatic imprinting since plants do not have germline. This long-term stress memory plays an important role in the adaptation and evolution of plants [145]. Epigenetic regulation of genes depends on the types of epigenetic marks and their position on genes [146]. In plants, DNA methylation happens in diverse types of cytosine contexts, including CpG, CpHpG, and CpHpH (where H represents A, T, or C); methylation at these sites is catalyzed by METHYLTRANSFERASE 1 (MET1), CHROMOMETHYLASE 3 (CMT3), and DOMAINS REARRANGED METHYLTRANSFERASE 2 (DRM2), respectively. In Arabidopsis, DNA methylation can be erased by members of the DNA glycosylase family of demethylases, including DEMETER (DME), ROS1, DEMETER-LIKE 1 (DML1), DML2, and DML3.

### 4.2. Histone Code

The N-terminal extremity of histone is known as the *tail* and is rich in basic amino acids such as lysine and arginine. In addition to DNA methylation, covalent modifications to histone tails, such as methylation, acetylation, phosphorylation, ubiquitination, sumoylation, glycosylation, and ADP-ribosylation constitute another type of epigenetic code, which is conserved across different kingdoms. Modifications to histone tails alter chromatin packaging with the consequence that condensed chromatin structure, such as in the B compartment, results in transcription inhibition because the transcription machinery is unable to access DNA, while loose chromatin, such as in the A compartment, results in transcription activation [147].

According to the histone code hypothesis, the transcription of genetic information encoded in DNA is in part regulated by chemical modifications carried by histone proteins on their unstructured ends (tail) [148]. Unlike acetylation, methylation shows a diverse pattern; several different arginine and lysine residues (R3 of H2A, R3, K20 of H4 and K4, K9, K27, K36, R2, and R17 of H3, etc.) can undergo various types of methylation such as mono, di or tri-methyl residues (arginine undergoes mono and di-methylation only while lysine can undergo mono, di and tri methylation). Depending on the type of methylation, histones can either activate or inhibit the expression of a gene. For example, H3K4 trimethylation activates transcription but K9 and K27 dimethylation in H3 acts as a transcription repressor. Methylation does not alter the net charge of histone-like acetylation but it affects hydrophobicity by the addition of methyl groups and hence may change histone DNA interactions or may create a binding site for various proteins that promote or inhibit the transcription machinery. Histone lysine methyl transferases (HKMT) and protein arginine methyl transferases (PRMT) are responsible for lysine and arginine methylation, respectively. As an example, CAM plants switch from the C3-photosynthetic cycle to the CAM pathway to reduce water loss in case of drought stress [149]. This switching process from the C3 to CAM pathway is coupled with the activation of genomic methylation and hypermethylation of satellite DNA [150].

### 4.3. Histone Modifications

ChIP-seq and spectrophotometer analyses of the four histone modifications, histone H3 lysine4 trimethylation (H3K4me2), H3K4me3, H3K9me2, or H3K27me3 in seedlings of Arabidopsis plants under salt and drought stress, revealed that the priming treatment (under which seeds are rehydrated and then dried back just before germination) altered the epigenomic landscape. After re-watering, leaf water potential, membrane stability, photosynthetic processes, reactive oxygen species (ROS) generation, anti-oxidative activities, lipid peroxidation, and osmotic potential completely recover in the case of moderately stressed plants but do not fully recover in severely stressed plants [151,152,153]. When rewatered, plants recover by growing new shoots. The extent of recovery due to rewatering strongly depends on drought intensity, duration, and plant genetics. In wheat, higher photosynthetic rates during drought and rapid recovery after rewatering produce less-pronounced yield declines in tolerant cultivars than in susceptible ones, which suggests that their ability to maintain functions during drought-stress and to quickly recover after rewatering are important factors determining their final productivity [65].

The output of gene transcription is influenced by the input of stress signals due to the well-documented trans-cis interaction between TFs and target DNA regions. However, there are still gaps between the transcription of genes and the anchoring of TFs, and these gaps in chromatin accessibility may be of fundamental importance. Histone methylation is one of the most functionally diverse epigenetic mechanisms because it can either exert activation or repression on target genes depending on the location of lysine residues, in contrast to DNA methylation or histone acetylation, which mark gene repression and activation, respectively [154]. Sani et al. [155] showed that priming-induced changes also depended on the specific lysine residues that were methylated. Despite being small, the changes were confirmed by semi-quantitative-PCR; they varied in number and preferentially targeted TFs. Several genes with priming-induced differences in H3K27me3 showed altered transcriptional responsiveness to a second stress treatment but the number of location-specific changes of H3K27me3 decreased, suggesting that the memory fades over time. In a genome-wide investigation of the gene expression profile in rice under drought-stress, differential H3K4me3 methylation was found to be significantly and positively correlated with transcript level only for a subset of genes under drought stress conditions. Moreover, for the H3K4me3-regulated stress-related genes, the H3K4me3 modification level was mainly increased in genes with low expression and decreased in genes with high expression under drought-stress [156].

Indeed, histone modifications, such as H3K4me3, H3K9ac, H3K9me2, H3K23ac, H3K27ac, H3K27me3, and H4ac, along with DNA methylation could be correlated with gene expression in response to abiotic stresses [157]. Some of the histone modifications occur quickly in response to environmental variations, while others occur gradually according to the challenges imposed on gene expression to control physiological homeostasis and development under environmental stress. Since histone acetylation tends to induce gene activation its removal can lead to gene repression and silencing. Although changes in histone modifications can be correlated with gene activity, the molecular mechanisms through which the chemical modifications influence the chromosomal structure and the accessibility of TFs are still not fully understood.

Plants memorize abiotic stress when exposed to it and can better tolerate it when experiencing it again later on [158]. The memorization process induced by DNA and histone modifications occurs through (i) the up-regulation of small RNAs (micro-RNAs or miRNAs) and short interfering RNAs (siRNAs), (ii) the down-regulation of repressors (specific proteins inhibiting transcription), and (iii) the down-regulation of specific siRNAs, which induce the up-regulation of activators required for the regulation of plant hormone and transcription factors. In Arabidopsis, in addition to siRNAs, it has been shown that lncRNA also participates in the regulation of plant stress-responsive gene expression via DNA de novo cytosine methylation through the RNA-directed DNA methylation (RdDM) pathway. RNAPIV-generated siRNAs could be loaded to Argonaute 4 (AGO4) and interact with lncRNAs generated by RNAPII to constitute a siRNA–AGO4–lncRNA silencing complex, which subsequently recruits the DMT domains rearranged methyltransferase 2 (DRM2) to mediate DNA de novo cytosine methylation. Mutants deficient in NRPD2, an essential subunit of RNAPIV, were hypersensitive to heat stress, suggesting that the RdDM pathway is essential to the regulation of plant stress responses (see refs in [159]).

Interestingly, the family of SWI2/SNF2 chromatin remodeling complexes can regulate the methylation of both DNA and histone marks for gene activation under environmental stresses [160], as was shown for drought tolerance in rice [161]. Examples of histone modifications and chromatin remodeling are (i) rice SWI/SNF2 ATPase BRHIS1 that regulates the expression of disease defense-related *OsPBZc* and *OsSIRK1* genes through specific interaction with mono-ubiquitinated *H2A.Xa/H2A.Xb/H2A.3* and *H2B.7* variants at those gene loci, (ii) Arabidopsis BRM that represses the expression of heat-activated *HSFA3* and *HSP101* genes by removing H4K16ac at their chromatin loci through interaction with HD2C, and (iii) PWR proteins that form a chromatin-remodeling complex with HDA9 and ABI4 to repress the drought-responsive *CYP707A1/2* genes in Arabidopsis. Histone modifiers and transcription regulators also coordinate chromatin dynamics and nucleosome configurations at transcription sites to modulate gene expression [162].

The interplay between histone modifications and DNA methylation provides plants with a multifaceted and robust regulatory circuitry for transcriptional reprogramming in response to stress. For example, DNA methylation changes and various histone modifications, such as H3K4me3, H3K9ac, and H3K9me2 are concertedly regulated for transcriptional activation or repression of the salt-responsive genes such as *Glyma08g41450*, *Glyma11g02400*, *Glyma20g30840* in soybean as well as *SUVH2/5/8*, *ROS1*, *MSH6*, *APUM3*, *MOS6*, and *DRB2* in Arabidopsis. The rice transcriptional complex SUVH7-BAG4-MYB106, consisting of a DNA methylation reader, a chaperone regulator, and a TF, activates *OsHKT1*. In addition, the expression of the Arabidopsis pathogenesis-related *SNC1* gene is cooperatively regulated by the chromatin-remodeling proteins CHR5 and DDM1/SYD for nucleosome occupancy and DNA methylation, along with the histone modifiers HUB1/2 and ATXR7/MOS9 for H2Bub and H3K4me3, respectively, in plant immune responses [163].

The HDA9-PWR-ABI4 complex plays a central role in the regulation of the molecular response to drought stress through the inhibition of ABA catabolism to promote the accumulation of active ABA, thereby protecting plants against dehydration. ABA regulates several physiological processes, including plant growth and development, flowering time as well as leaf size and morphology. Further investigations are required to understand how this complex modulates genes activated by ABI4 at a chromatin level [164].

## 5. Chromatin Structure

Chromatin immunoprecipitation or ChIP is a method used to investigate the dynamic interactions of specific proteins and DNA sites. These interactions have a significant role in various cellular processes such as replication, transcription, DNA damage repair, genome stability, and gene regulation [165]. This technique allows the study of various cellular mechanisms inside cells according to protein-DNA interactions. As the name chromatin immunoprecipitation suggests, this method allows the identification of DNA-protein interactions by immunoprecipitation. ChIP is being extensively used to depict TFs, variants of histone, chromatin-modifying enzymes, and histone post-translational modifications [166,167]. ChIP followed by high-throughput sequencing (ChIP-seq) is used to study genome-wide protein-DNA interactions to understand gene regulation in native chromatin. ChIP-seq is used to identify, map, and characterize the specific DNA fragments that interact with proteins in vivo [168].

**Figure 3:**
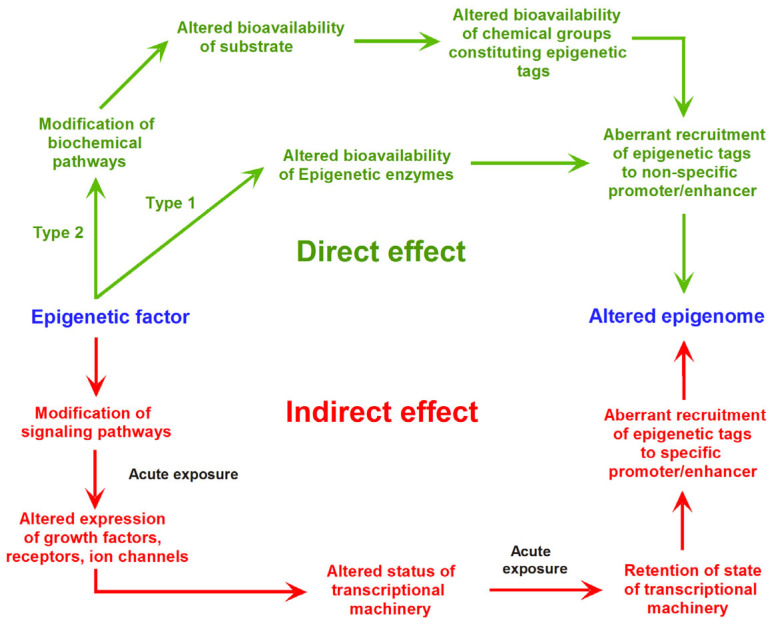
Ways that an epigenetic factor might affect the epigenome and change how genes are expressed. Direct and indirect influences on the epigenome might result from epigenetic changes produced by an external source or intrinsic environment. Red is for epigenetic factors with indirect impacts, while green is for factors with direct effects. Here, epigenetic factor is understood as any molecule, such as ncRNAs, metabolites, or phytohormones induced by endogenous or environmental stresses, able to induce global changes affecting the DNA methylation of multiple genes or modify the expression of specific genes (adapted from ref. [169], Copyright 2014 owned by Frontiers. More details on “Copyright and Licensing” are available via: https://creativecommons.org/licenses/by/4.0/).

ChIP assays have been successful in identifying the histone modifications responsible for epigenetic regulation. However, the results obtained by ChIP assays provide only indirect candidate residues that may be targets of modifications [136]. Thus, gathering direct or indirect pieces of evidence that enable correlation of the functions of histone modifications with the plant epigenetic landscape is still a challenge, as shown in Figure 3 [169].

The mapping of chromatin by CHIP-seq in seedlings of allohexaploid wheat enabled detection of distinct chromatin architectural features surrounding various functional elements including genes, promoters, enhancer-like elements, and transposons [170]. Thousands of new gene regions, trans- and cis-regulatory elements were identified based on the combinatorial pattern of chromatin features. As expected, the subset of genome active regulatory elements that include promoters and enhancer-like elements (roughly 1.5%) is characterized by a high degree of chromatin openness, histone acetylation, abundance of CpG islands, and low DNA methylation levels since they are associated with genes, which are found in their majority in the A compartment [147]. A comparison across sub-genomes revealed that negative selection is targeting sequence and chromatin features involved in gene regulation. The divergent enrichment of cis-elements between enhancer-like sequences and promoters implies that these functional elements are targeted by different TFs [170].

In mammals, chromatin analyses revealed that: (i) low recombination domains and regions of elevated linkage disequilibrium (LD) tend to coincide with topologically associated domains (TAD) and isochores; (ii) double-strand break (DSB) and recombination frequencies increase in the short loops of GC-rich TADs, whereas recombination cold spots are typical of lamina-associated domains (LAD), which mediate chromatin tethering to the lamina of the nuclear envelope; and (iii) binding and loading of proteins, which are critical for DSB and meiotic recombination, are higher in GC-rich TADs. Recombination is favored or suppressed in specific mega-base-sized regions [171] according to linkage disequilibrium (LD), which is positively correlated to GC%, with strong LD being typical of GC-poor regions [172]. In humans, hotspots of recombination are associated with local GC spikes (1 to 2 kb in size) and with an increase of the local mutation rate resulting in G or C nucleotides, but without an effect on substitution rate or divergence [173], which means that only allele interchanges between sister chromatin are affected. Thus, GC-rich R-bands favor DSB due to lower chromatin fiber stiffness [174]. As a result, TADs and LADs are characterized by a non-random association of chromosomal recombination profile and chromatin architecture. TADs represent an assembly of chromatin loops (with boundaries of 0.2–2 Mb in size [175], which can be resolved into contact domains, of 185 Kb in median size [176]. LADs have a median size of 500 Kb, are GC-poor, and are distributed over the whole genome [177,178,179]. DSBs correlate with H3K4me3 [180,181] and typically take place in GC-rich nucleosome-depleted loop sequences with the consequence that GC-rich domains are more recombinogenic compared to GC-poor ones. GC-rich regions are typically (i) highly transcriptionally active, (ii) characterized by H3K4me3, H3K9ac, and H3K36me3 marks, and (iii) harbor shorter loops than GC-poor domains [179]. The correspondence between LD-regions, isochores, and TADs boundaries strongly suggests the existence of a common genomic code of chromatin architecture in mammalian meiotic and mitotic cells [182].

In plants, chromatin functionalities occurred through DNA mechanical anchoring via nucleolus-associated domains (NADs), which are chromatin regions interacting with the nucleolus, and LADs. Chromosomal A and B compartments can be further segmented as TADs of intertwined loops in the range of 0.1–1.0 Mb of relatively independent chromatin regions, which promote a larger rate of interaction between cis and trans-regulatory sequences (enhancers and promoters) as well as gene kissing [183,184]. TADs were only observed in plant species having genome sizes larger than 400 Mb [170,185,186]. Chromatin loops connect the distant regulatory elements to their target loci by physical approximation. The regulatory function of chromatin loops occurs through the formation of (i) a repressive loop by the intermediation of histone modifiers−H3K27me3−polycomb protein−lncRNAs complex, such as in heterochromatin, which is characterized by a low gene density, low transcriptional activity, high repressive epigenetic modifications, and higher transposon density (B compartment); (ii) a silencing loop by the intermediation of H3K9me2-reader (ADCP1)−ncRNAs complex, and (iii) a transcriptional loop hub formed by the intermediation of the H3K4me3 modifiers-RNA Pol-II-eRNAs complex and characterized by high gene density, activating epigenetic modifications, and active transcriptional activity (A compartment) [161,187].

A general model for the role of epigenetic elements in stress responses of wheat and barley has been proposed by Kong et al. [159] where DNA methylation, histone modifications, and chromatin remodeling are regulated through siRNA and lncRNA epigenetic processes, including RdDM and histone modification as well as genome topology changes, affecting gene expression in response to the environmental stresses.

## 6. Transcription Factors

TFs help plants to respond to abiotic stress through priming stimuli by inducing the accumulation of inactive transcription factors, and inactive signaling compounds. A primed state is a kind of state of readiness for another stress event by which plants are capable of quicker and more effective activation of stress-protective responses (see refs in [188]). TFs are the endpoints of signal transduction networks and may control numerous pathways simultaneously, which has prompted practical strategies for engineering plants with improved stress tolerance [189,190]. Many key genes and transcription regulators governing morpho-physiological traits were found to control root architecture and stomatal development for soil moisture extraction and its retention, which justifies their use as targets for molecular breeding strategies of selective breeding for drought tolerance [191]. For example, *DRO1* in rice (*Oryza sativa* L.) and *ERECTA* in Arabidopsis and rice were identified as enhancers of drought tolerance via regulation of root traits and leaf transpiration. Tools such as (i) the reference genome sequence for wheat, (ii) functional reverse genetics, and (iii) genome editing technologies are expected to aid in deciphering the functional roles of genes and regulatory networks underlying adaptive phenological traits as well as the development of drought-tolerant cultivars [192]. Another example is *DUO-B1*, which is a gene encoding an APETALA2/ethylene response factor (AP2/ERF) protein, one of the largest families of transcription factors that have been linked to a variety of biological processes. Based on the DNA binding domain (DBD), AP2/ERFs were classified into AP2, RAV (related to *Abscisic acid insensitive3/Viviparous1*), DREB (subgroup A1–A6), ERF (subgroup B1–B6), and others [193]. The members of the RAV (one of the most abundant transcription factor families in plants) and ethylene response (ERF) subfamily are frequently engaged in phytohormone signaling, disease resistance, and abiotic stress response. CRISPR-Cas9 genome editing showed that the wild gene controls meristem activity and branching in its orthologues from *Brachypodium distachyon* (L.) P. Beauv., bread wheat, and Arabidopsis. CRISPR-induced mutations of *DUO-B1* led to mild supernumerary spikelets, increased grain number per spike, and increased yield under field conditions without affecting other major agronomic traits. Pyramiding this gene with loci that are responsible for improved photosynthesis and nitrogen use efficiency could broaden the utilization of *DUO-B1* alleles to increase yield potential [194].

It has been found that genes involved in drought tolerance were regulated by TFs such as: (i) Basic leucine zipper (bZIP), which controls various biological functions involved in pathogen defense, seed maturation, light and stress signaling, and flowering [195]; (ii) Basic helix-loop-helix (bHLH) TFs, which act as transcriptional regulators involved in stress response, phytochrome signaling, anthocyanin biosynthesis, fruit ripening, carpel and epidermal development [195]; (iii) WRKY, which has a ~60 amino acid domain whose WRKYGQK sequence is completely conserved and followed by a zinc finger motif, participating in signaling networks for plant defense (some WRKY family members may play important roles in the maturation of root cells, senescence, dormancy, fruit maturity, tannin synthesis in the seed coat, and embryo development [196]); (iv) Myeloblastosis (MYB), which is involved in the regulation of primary and secondary metabolism, the control of cell development and the cell cycle as well as in the participation in defense and response to various biotic and abiotic stresses, hormone synthesis, and signal transduction [195]; and (v) NACs, which have a variety of significant functions in plant development as well as in responses to biotic (defense to parasites) and abiotic stresses. The establishment of shoot apical meristems, lateral root development, senescence, cell wall construction, and secondary metabolism are other processes that NACs control in plants [195].

## 7. Transgenic Approach

The main response mechanisms to drought stress were uncovered by studying *A. thaliana*, and multiple drought tolerance genes that are highly conserved among plants are being engineered into crops [197]. These genes relate to drought escape, control of flowering time, stomatal responses, T6P pathways, and some root traits. So far, most plants with enhanced drought resistance have displayed reduced crop yield. However, the uncoupling of drought tolerance and plant growth seems possible at least in Arabidopsis since it was shown that increasing brassinosteroid receptors in vascular plant tissues confers tolerance to drought without penalizing growth [198]. Arabidopsis is a good model to test drought-responsive strategies that may have interesting agronomic potential and might be translated into crops by transformation. Of course, this issue must be carefully addressed since genes from different plant species may not behave in the same way from one system to the other. One obvious example is that the GC content of an Arabidopsis gene will be below that of the grass genome context and would probably end up methylated. The question here is not merely to transfer a gene as it is but rather to transfer the functional information it carries.

Plants exposed to abiotic stress exhibit enhanced levels of ROS, which affect chlorophyll biosynthesis, photosynthetic capacity, carbohydrate, protein, lipid, and antioxidant enzyme activities. Thus, transgenic breeding offers a tempting platform to mitigate these negative effects [199].

However, pyramidizing genes by transgeny may be difficult because stress tolerance phenotypes are QTLs. They must first be dissected to exploit their use by accelerating genetic introgression using molecular markers or site-directed mutagenesis [200].

Genetic improvement for drought-tolerant wheat cultivars is a major aim of wheat breeders as indicated in Table S3. This table summarizes the current genes used to improve the drought tolerance of wheat through genetic engineering [201].

## 8. Epigenetics and Crop Improvement

As seen above, epigenetics is another layer of information enabling an organism to adapt to environmental conditions such as biotic and abiotic stresses. This adaptation occurs by modulating massive gene expression through hundreds of molecular players including ncRNAs [202,203]. In this way, epigenetic changes act as a buffer mechanism to alleviate short term stressful effects from the environment by adapting the organism’s gene expression response [204]. When stress conditions last for a long time, the gene expression profile may, eventually, be passed on to the next generation, but the benefits could be at the expense of undesirable phenotypic traits as well [205], which means that the mechanism involved must be well understood. Both short and long-term memory has specific advantages and drawbacks that favor selective breeding to produce so-called smart-crops [206]. However, a difficulty with epigenetics is that the effect it induces can be confused with the genetic trait that supports it. Hofmeister et al. [207] developed an epigenotyping procedure that enabled uncoupling this type of interaction, which allows a better understanding of patterns of epiallele inheritance. In Arabidopsis, exposure to abiotic stress during several generations was observed to induce heritable phenotypic changes, but the observed effects depended on plant genotype, which suggested an interaction between genetic background and inheritance of induced epigenetic patterns [208]. Since the experiment involved several generations, allele shuffling must have occurred from one generation to the next and even under the hypothesis that the epigenetic pattern would be the same, different phenotypes would result because of different allele combinations being expressed. Nonetheless, there is no reason to think that it is not possible to select alleles for the epigenetic (epialleles) process itself and this could result in different epigenetic patterning. Surveys have shown that stable epialleles can accumulate, segregate in populations, and be selected for [209,210,211]. Stable epialleles can therefore be linked to quantitative trait loci (epiQTL). To identify epiQTLs, epigenetic recombinant inbred lines (epiRILs) are created using genetically identical parental (isogenic) plants, which differ in levels of DNA methylation [207]. Epigenetic traits that could contribute to improving drought tolerance were reviewed in [206] and could be obtained by recurrent epi-selection, hybrid mimics, epigenomic selection, epigenome editing, and stress priming mechanisms.

Recently, the msh1 mutant was discovered to induce epigenetic reprogramming. The MSH1 is a plant-specific gene which encodes a protein that targets plastids. Disruption of MSH1 function leads to variation in plant growth rate, flowering time, response to short day length, leaf morphology, variegation, and stress response, which are phenotypes that are reproducible across a range of plant species [212]. The msh1 mutant induces genome-wide DNA methylation repatterning, changes in siRNA expression, and heritable nongenetic memory. Gene networks affected by methylation repatterning include auxin-related pathways, so that altered expression of auxin-response genes contributes to the increased plant vigor phenotype in a way similar to heterosis [213].

As an example of an MSH1 system application for the induction of epigenetic variation in crops, Raju et al. [214] developed epi-lines by crossing a wild type with an isogenic transgenic soybean line for msh1. The progeny showed a wide variation both in greenhouse and field trials and a low epitype–environment interaction, which indicated yield stability and low effect of environmental constraints.

One may conclude from the experiments with msh1 that involvement of a significant amount of individual RNA and proteins seems to be essential for manifesting phenotypic diversity and phenotypic expression of heterosis and hybrid vigor. According to Bharti Thapa and Shrestha [215], the best hybrids seem to have a significantly larger number of gene expressed compared to the best parent at different stages. Concerning methylation level, it was found that (i) it is lower in heterotic hybrids than in related non-heterotic hybrids, (ii) it is higher in old, low yielding inbred lines, and (iii) it is lower in modern inbred lines, especially those selected for high yield [215].

As seen above, plant germ lines develop from somatic cells [216], which means that epigenetic information can be transferred by vegetative cloning, graft, or through gametogenesis, but epigenetic states can often be unstable in this last case. Vegetative cloning and graft are not feasible in cereals, but apomixis seems to be a viable option by enabling F1 hybrids with superior traits to be clonally propagated. Engineering synthetic apomixis was performed in inbred rice by converting the meiotic division into mitotic division and triggering parthenogenesis. A hybrid apomictic clone that produces more than 95% of clonal seeds across at least three generations was produced from a commercial F1 rice hybrid by T-DNA transformation of embryo-derived callus [217]. This achievement combined with the msh1 mutation opens the way to induce and stabilize epigenomes in crops, which offers the potential of accelerating selective breeding for drought tolerance in arid and semi-arid regions.

## 9. Conclusions

Understanding the physiological processes that lead to increased drought tolerance in plant models such as Arabidopsis, rice, and maize is helping to map the genes that can be validated as functional for selective breeding purposes. Drought tolerance is a QTL, but some of the genes involved in that QTL usually explain a larger proportion of the detected phenotype. Genetic and physical mapping through SNP association and GWAS helps to detect these genes, which can then be proven to be involved in drought tolerance by functional assays such as knockdown. Such genes can then be transferred by transgenesis or by selective breeding. According to the classical approach, in vitro culture and transgenesis help to produce clones carrying specific traits that can then be crossed with elite lines for pyramiding genes. Nonetheless, improving the understanding of epigenetics and stress memory will also contribute to the selective breeding of complex agronomic traits. The uncovering of DNA-binding sites of particular binding proteins, such as transcription factors or chromatin-associated proteins is enabling the exploration of the genome epigenetic landscape for such purposes. However, epigenomes are known to be unstable from one generation to the next, which makes their industrial exploitation difficult. Recently the discovery of the msh1 mutation has made it possible to produce epigenetic variability in several crops with similar effects to heterosis. New techniques of synthetic apomixis are being developed that permit clonal propagation accessions for several generations, which enable us to glimpse the future contribution of epigenetics to selective breeding including for drought tolerance.

## Data Availability

Not applicable.

## Supplementary Materials

The following supporting information can be downloaded at: https://www.mdpi.com/article/10.3390/plants12112170/s1, References [218,219,220,221,222,223,224,225,226,227,228,229,230,231,232,233,234,235,236,237,238,239,240,241,242,243,244,245,246,247,248,249,250,251,252,253,254,255,256,257,258,259,260] are cited in the supplementary materials. Table S1a: QTLs of agronomic traits identified for drought tolerance in cereals (updated from [29]); Table S1b: QTLs of agronomic traits identified in soybean for drought tolerance (updated from [228]); Table S2: Drought stress and their related candidate genes in model plants (updated from [29]); Table S3: Improving drought tolerance of cereals through engineering genes (updated from [201]).
