# Supplementary material for: Plant Tolerance to Drought Stress with Emphasis on Wheat
Source: Plants (Basel). 2023 May 30;12(11):2170. doi: 10.3390/plants12112170 (PMC10255504; doi:10.3390/plants12112170)
Supplement: Supplementary file 1 [file plants-12-02170-s001.zip › TableS1.pdf]

Table S1a. QTLs of agronomic traits identified for drought tolerance in cereals. A complement of this table is available from [29].

Traits	QTL number	Linkage group	Crop	Phenotype variation, %	References
Grain volume weight	5	2A, 2B, 5A, 5D, 7B	Wheat	14.9-24.5	[218]
Days to heading	4	4A, 5A, 5D, 7B	Wheat	13.4-38.4	[218]
Days to flowering	5	1, 2, 3, 5, 11	Rice	*3.2-11.1*	[219]
Plant height	2	2D, 6A	Rice	11.4, 17.2	[218]
	2	1, 11	Rice	6.0, 7.7	[219]
	1	1	Rice	13.1	[220]
	2	1, 6	Rice	19.5, 25.0	[221]
	1	10	Maize	10.0	[222]
	3	4H, 5H	Barley	5.1-7.7	[223]
Leaf number	2	3, 5	Barley	2.6, 3.3	[224]
Leaf weight	3	2, 4, 7	Barley	2.9-3.6	[224]
Stomata number	1	1	Barley	16.5	[224]
Leaf rolling index	7	1, 2, 3, 4, 7, 12	Rice	4.0-13.8	[225]
Leaf withering degree	6	1, 2, 3, 5, 6, 10	Rice	4.2-11.1	[225]
Chlorophyll content	4	1, 3, 5, 11	Rice	3.6-6.7	[225]
Days to 50% flowering	3	6	Rice	2.6-6.0	[220]
Tillers per plant	1	11	Rice	19.5	[221]
Culm length	1	2	Rice	29.5	[221]
Leaf dry weight	1	3	Rice	34.0	[221]
Stem dry weight	1	3	Rice	7.5	[221]
Total shoot dry weight	1	3	Rice	6.9	[221]
Total water uptake	2	3, 4	Rice	10.0, 17.1	[221]
Root weight	2	1, 2	Barley	11.2, 14.5	[224]
Total root dry weight	1	3	Rice	20.9	[221]
Deep root length	3	3, 9, 11	Rice	3.8-29.5	[221]
Deep root volume	1	3	Rice	25.8	[221]
Deep root surface area	2	3	Rice	13.6, 32,1	[221]
Deep root diameter	2	3	Rice	18.3, 25.4	[221]
Ear height	4	4, 6, 7	Maize	6.0-13.0	[226]
Ear weight	5	1, 4, 5, 9	Maize	4.8-15.6	[226]
Ear length	3	2, 4, 9	Maize	6.4-14.5	[226]
	5	2, 3, 8, 10	Maize	3.5-8.8	[227]
Ear diameter	5	3, 4, 5, 7	Maize	2.0-3.2	[227]
Ear height-to-plant height ratio	5	1, 3, 6, 8	Maize	4,1-11.2	[222]
Grain weight per ear	9	1, 2, 4, 6, 8, 9	Maize	2.8-12.9	[222]
Number of rows per ear	5	1, 5, 10	Maize	9.0-12.7	[227]
Number of kernels per row	6	3, 4, 5, 7	Maize	1.6-13.2	[227]

Kernel ratio	6	1, 3, 6	Maize	4.1-13.6	[222]
Anthesis-silking interval	2	4, 7	Maize	6.4-8.5	[226]
Cob weight	4	1, 4, 9	Maize	4.0-12.4	[226]
100-kernel weight	2	1, 4	Maize	5.1-13.1	[226]
1000-kernels weight	5	1, 2, 7, 10	Maize	2.3-34.2	[227]
	3	2, 4	Barley	5.2-5.7	[223]
Kernel length	2	5, 7	Maize	10.2-32.1	[227]
Kernel width	6	1, 2, 6, 7	Maize	2.0-5.0	[227]
Kernel thickness	5	2, 5, 7, 8, 10	Maize	2.5-4.0	[227]
Kernel per spike	8	2	Barley	5.1-5.7	[223]
Dry weight in tillering	2	4	Barley	6.3-6.6	[223]
Biomass	4	2, 5	Barley	5.0-6.7	[223]
Hectoliter weight	3	1, 5	Barley	7.2-11.0	[223]
Harvest index	3	1, 5	Barley	5.8-11.0	[223]

Table S1b. QTLs of agronomic traits identified in soybean for drought tolerance. A complement of this table is available from [228].

QTL Name	Traits	Chromosome and linkage group	Phenotypic variation, %	**References**
Gm05_2548644	SW	5	10.4	[229]
Gm06_43432590	SW	6	29.6	[229]
Gm09_36869408	SW	9	8.0	[229]
Gm10_44445941	SW	10	20.0	[229]
Gm12_37803362	SW	12	7.8	[229]
Gm19_45553057	SW	19	9.3	[229]
qMRL001	MRL[1]	4	26.0	[30]
qMRL002	MRL	7	33.0	[30]
qMRL003	MRL	12	17.0	[30]
qLRN001	LRN[2]	(11)	39.0	[30]
qBRT001	BRT[3]	11	12.0	[30]
qRFW001	RFW[4]	1	26.0	[30]
qRFW002	RFW	(2)	20.0	[30]
qRDW001	RDW[5]	(2)	34.0	[30]
qRDW002	RDW	12	19.0	[30]
qSDW001	SDW[6]	2	34.0	[30]
qSDW002	SDW	10	18.0	[30]
qSDW003	SDW	12	18.0	[30]
qSFW001	SFW[7]	(2)	33.0	[30]
qSFW002	SFW	6	29.0	[30]
qSFW003	SFW	8	12.0	[30]
qSFW004	SFW	12	21.0	[30]
qSFW/SDW001	SFW/SDW[8]	3	19.0	[30]
qSFW/SDW002	SFW/SDW	5	20.0	[30]

qSFW/SDW003	SFW/SDW	9	15.0	[30]
LA-D	LA[9]	18	6.3	[47]
RECL-D	RECL[10]	15	9.2	[47]
RWCL-D	RWCL[11]	16	10.1	[47]
PH-D	PH[12]	13	5.9	[47]
NNMS-D	NNMS[13]	16	15.2	[47]
BN-D	BN[14]	11	5.3	[47]
BIOMASS-D	BIOMASS[15]	11	7.6	[47]
SWPP-D	SWPP[16]	2	4.5	[47]
SWPP-D	SWPP	8	5.1	[47]

[1]MRL: Maximum root length; [2]LRN: Lateral root number; [3]BRT: Basal root thickness; [4]RFW: Root fresh weight; [5]SDW: Shoot dry weight; [6]SFW: Shoot fresh weight; [7]SDW: Shoot dry weight; [8]SFW/SDW: Ratio of shoot fresh weight to shoot dry weight; [9]LA: Leaf area; [10]RECL: Relative electric conductivity of leaves; [11]RWCL: relative water content of leaves; [12]PH: Plant height; [13]NNMS: node number of main stem; [14]BN, branch number; [15]BIOMASS: Biomass; [16]SWPP, seed weight per plant.
