# Supplementary material for: Plant Tolerance to Drought Stress with Emphasis on Wheat
Source: Plants (Basel). 2023 May 30;12(11):2170. doi: 10.3390/plants12112170 (PMC10255504; doi:10.3390/plants12112170)
Supplement: Supplementary file 1 [file plants-12-02170-s001.zip › TableS2.pdf]

Table S2. Drought stress and their related candidate genes in model plant. A complement of this table is available from [29].

Gene	Acession number	Plant species	Function	References
IDM1	AT3G14980.1	*T. aestivum*	Preventing DNA hypermethylation of highly homologous multicopy genes and other repetitive sequences.	[52]
CIPK3	AT2G26980	*T. aestivum*	Encodes a serine-threonine protein kinase whose expression increases in response to ABA, cold, drought, high salt, and wounding conditions. The gene is expressed in developing seeds and seedlings	[52]
PAL4	AT3G10340	*T. aestivum*	Controls carbon flux to a variety of bioactive small-molecule aromatic compounds, and lignin	[52]
CYP73A5	AT2G30490	*T. aestivum*	Catalyzes the first oxidative step of the phenylpropanoid pathway in higher plants by transforming trans-cinnamate into p-coumarate	[52]
ABCG11	AT1G17840	*T. aestivum*	Essential for synthesis of plant organs like root development	[52]
WAKL21	A4A49_34180	*T. aestivum*	Signaling receptor of extracellular matrix component	[52]
AMY1	AT4G25000	*T. aestivum*	Expressed 7 to 9 days after from pollination and during the seed development	[52]
RPS15AE	AT4G29430	*T. aestivum*	Enables structural constituent of ribosome	[52]
CYP94C1	AT2G27690	*T. aestivum*	Bioactive phytohormone of the jasmonate hormonal pathway regulating important defensive and developmental processes in plants	[52]
NPY1	YGL067W	*T. aestivum*	Regulates phototropic responses along with protein kinases	[52]
PSAT2	AT2G17630	*T. aestivum*	Enables O-phospho-L-serine:2-oxoglutarate aminotransferase activity, pyridoxal phosphate binding	[52]
FH6	AT5G67470	*T. aestivum*	Enables actin binding	[52]
RXF12	AT1G58370	*T. aestivum*	Improves the dough's rheological properties, such as softness, ductility, and elasticity, and the bread's specific volume and crumb hardness	[52]
TIN1	AT5G64510	*T. aestivum*	Acts as transcriptional factor which controls buds outgrowth	[52]
FY	AT5G13480	*T. aestivum*	Regulation of flowering time	[51]
MAP1A	AT2G45240	*T. aestivum*	Removes the N-terminal methionine from proteins leading to abnormal development	[51]
LRL2	AT4G30980	*T. aestivum*	Regulates root hair and sperm cell	[51]

			development	
ERMO2	AT3G07100	*T. aestivum*	Required for endoplasmic reticulum (ER) morphology	[51]
RGLG2	AT5G14420	*T. aestivum*	Negatively regulates the drought stress response by mediating the ERF53 transcriptional activity	[51]
RBOHB	AT1G09090	*T. aestivum*	Plays a role in seed after-ripening	[51]
GNL1	AT5G39500	*T. aestivum*	Required for endoplasmic reticulum (ER) morphology	[51]
CYP70982	NA	*T. aestivum*	Not found	[51]
AGO1	AT1G48410	*T. aestivum*	Regulates genes involved in the cross talk between auxin and light signaling during adventitious root development	[51]
DMP10	OSNPB_010389700	*T. aestivum*	DOMAIN MEMBRANE PROTEIN 10	[51]
PSKR1	AT2G02220	*T. aestivum*	Contains dual guanylate cyclase and kinase catalytic activities that operate in vivo	[51]
CSC1	AT4G22120	*T. aestivum*	Calcium-permeable stretch activated cation channel.	[51]
GME	AT5G28840	*T. aestivum*	Catalyzes the conversion of GDP-D-mannose to GDP-L-galactose.	[51]
ARR12	HvARR12	*H. vulgare*	two-component response regulator	[230]
PIP	HvPip	*H. vulgare*	Proline iminopeptidase	[230]
DI19-2	HvDI19-2	*H. vulgare*	Dehydration stress-inducible gene	[230]
ADC	HvADC	*H. vulgare*	Arginine decarboxylase	[230]
HRGP	HvHRGP	*H. vulgare*	Hydroxyproline-rich glycoprotein	[230]
PRP	HvPRP	*H. vulgare*	Proline-rich family protein	[230]
C4H	HvC4H	*H. vulgare*	Cinnamate-4-hydroxylase	[230]
OsALDH2–1	Os01g40860	*O. sativa*	Expressed in seeds and no essential for seed vigor	[231]
OsALDH2–2	Os01g40870	*O. sativa*	Expressed in seeds and essential for seed vigor	[231]
OsWRKY45	Os05g25770	*O. sativa*	Defense ABA signaling pathway	[231]
OsMSOD1	Os05g25850	*O. sativa*	Limits reactive oxygen species damage during drought stress	[231]
OsRCN1	Os11g05470	*O. sativa*	Expressed in vein leaf and important for floral transition	[231]
OsbZIP79	Os11g05480	*O. sativa*	Regulation of phytoalexin production	[231]
OsbZIP80	Os11g05640	*O. sativa*	Dehydration stress-inducible gene	[231]
LOC103637669	Zm00001d029938	*Z. mays*	Protein ARABIDILLO 1	[232]
S1_93277641	Zm00001d029937	*Z. mays*	Glycoprotein	[232]
PZE-103003226	Zm00001d039319	*Z. mays*	Tic22-like family protein	[232]
LOC100500952	Zm00001d042997	*Z. mays*	HIT-type zinc-finger family protein	[232]
LOC100284853	Zm00001d029937	*Z. mays*	Glycoprotein	[232]
LOC103637669	Zm00001d029938	*Z. mays*	Protein ARABIDILLO 1	[232]
LOC100272678	Zm00001d020506	*Z. mays*	26S proteasome non-ATPase regulatory subunit 9	[232]
LOC100283961	Zm00001d013992	*Z. mays*	Pyridoxal phosphate-dependent transferase family protein	[232]
chr2.S_68691618	Zm00001d003939	*Z. mays*	11-ß-hydroxysteroid dehydrogenase	[232]

S2_226449870	GRMZM2G070937	*Z. mays*	Leu-rich repeat protein kinase family	[232]
LOC100286093	Zm00001d012167	*Z. mays*	Silk fibroin (SF16) protein	[232]
SbZfl1	NA	*S. bicolor*	SET binding factor 1	[67]
SbCN12	NA	*S. bicolor*	Ortholog of ZCN12 that have been proposed to encode florigens	[67]
BADH	I8G32_RS03395	*S. bicolor*	2-hydroxycyclohexanecarboxyl-CoA dehydrogenase	[77]
RuBisCO	Csa_5G609710	*S. bicolor*	Ribulose bisphosphate carboxylase small chain, chloroplastic-like	[77]
PEPC	AT3G14940	*S. bicolor*	Phosphoenolpyruvate carboxylase	[77]
DHN	SORBI_3009G116700	*S. bicolor*	Dehydrin DHN1	[77]
PTKs	NA	*S. bicolor*		[77]
BADH	NA	*Brassica napus*	Betaine aldehyde dehydrogenase	[233]
P5CS2	AT3G55610	*B. napus*	Delta 1-pyrroline-5-carboxylate synthetase	[233]
ADC2	AT4G34710	*B. napus*	Arginine decarboxylase	[233]
btg-26	HID58_028326	*B. napus*	Aldehyde dehydrogenase family 7 member A1-<br>Brassica turgor gene 26	[233]
MDA	GRMZM2G081380	*Maize*	Ca^{2+} binding actin bunding protein	[234]
REC	GRMZM2G171179	*Maize*	AP2/EREB160 transcription factor	[234]
SOD	GRMZM2G152278	*Maize*	Calcium-dependent lipid binding protein	[234]
BART1_0-P29927	HORVU4Hr1G060370	*Barley*	Protein phosphatase 2C family protein	[235]
BART1_0-P34164	HORVU5Hr1G027910	*Barley*	Chaperone protein htpG family protein	[235]
BART1_0-p07678	HORVU2Hr1G010990	*Barley*	Aquaporin-like superfamily protein	[235]
BART1_0-P51213	HORVU7Hr1G037080	*Barley*	Low quality protein: annexin D2	[235]
BART1_0-P29181	HORVU4Hr1G049500	*Barley*	Sucrose synthase 3	[235]
BART1_0-P15058	HORVU2Hr1G106880	*Barley*	Putative zeaxanthin epoxidase	[235]
BART1_0-P37103	HORVU5Hr1G070720	*Barley*	Molecular chaperone HtpG	[235]
GhHUB2	GhHUB2-A (XM_016856414), GhHUB2-D (XM_016843047)	*Cotton*	Histone H2B monoubiquitinatin E3 ligase encoding gene	[236]
GhMKK3	HQ828070	*Cotton*	Enhanced drought tolerance	[237]
GhMAP3K40	LOC107939304	*Cotton*	Salt and drought stress tolerance at the germination stage	[238]
GhMPK4	CotAD_19088	*Cotton*	Increased sensitivity to ABA, salt, and drought	[239]
GhMPK17	NM001035863	*Cotton*	Osmotic and salt stress tolerance	[240]
GbMPK3	NP_190150	*Cotton*	Enhanced oxidative and drought stress tolerance	[241]
