# Supplementary material for: Plant Tolerance to Drought Stress with Emphasis on Wheat
Source: Plants (Basel). 2023 May 30;12(11):2170. doi: 10.3390/plants12112170 (PMC10255504; doi:10.3390/plants12112170)
Supplement: Supplementary file 1 [file plants-12-02170-s001.zip › TableS3.pdf]

Table S3. Improving drought tolerance of wheat through engineering gene. A complement of this table is available from [201].

**Gene**	**Source**	**Improved traits**	**References**
P5CS	*T. aestivum*	Promotes proline accumulation	[242]
TaFER-5B	*T. aestivum* cv TAM107	Promotes higher leaf's iron and ROS content, drought and temperature tolerance	[201]
TaPEPKR2	*T. aestivum* cv TAM107	Promotes higher drought tolerance and root length	[201]
TaSHN1	*T. aestivum* cv RAC875	Promotes lower stomatal density and leaf water loss, beter recovery after severe drought	[243]
TaNF-YB4	*T. aestivum* cv RAC875	Promotes more spikes	[243]
DREB/CBF	*T. aestivum* cv RAC875	Enhanced ability to survive to frost and drought	[243]
TuNAC69	*T. aestivum*	Larger root biomass and root length	[244]
TabZIP2	*T. aestivum* cv RAC875	Fewer spikes and seeds, higher seed weight	[243]
DREB	*T. durum* cv Langdon	Higher survival rate, slower growth, delayed flowering, lower grain yield	[245]
RRF	Rice	Promotes chloroplast development and eary stages of embryo development.	[246]
Rhodanese	Rice	Promotes cell cycle control by phosphatase superfamily protein	[246]
MRL1	Rice	A conserved pentatricopeptide repeat protein required for stabilization of rbcL mRNA	[246]
PGRL1B	Rice	A transmembrane protein present in thylakoids. Plants lacking PGRL1 show perturbation of cyclic electron flow	[246]
LHCB3	Rice	A component of the main light-harvesting chlorophyll a/b-protein complex of Photosystem II (LHC II)	[246]
RPH1	Rice	A chloroplast protein RPH1 (resistance to Phytophthora 1) involved in immune response to *Phytophthora brassicae*	[246]
APL1	Rice	Large subunit of ADP-glucose pyrophosphorylase, which catalyzes the first and rate-limiting step in starch biosynthesis.	[246]
HAD	Rice	Haloacid dehalogenase-like hydrolase (HAD) superfamily protein	[246]
SOQ1	Rice	Suppressor of quenching 1 that prevents the formation	[246]

		of a slowly reversible form of antenna quenching, thereby maintaining the efficiency of light harvesting.	
PEROXIN11C	Rice	Member of the *peroxin11* (*PEX11*) gene family, integral to peroxisome membrane, controls peroxisome proliferation	[246]
HvAKT2	Barley	Found in plasma membrane, H^+ homeostasis, K^+ uptake	[247]
HvHAK1	Barley	Found in plasma membrane, H^+ homeostasis, K^+ uptake	[247]
BART1_0-P34164	Barley	Molecular chaperone HtpG, stabilize the folding, association and translocation	[235]
BART1_0-p07678	Barley	Aquaporin protein that transport water	[235]
BART1_0-P47576	Barley	Betaine aldehyde dehydrogenase; it produces glycine betain and acts as osmoprotectant	[235]
BART1_0-P13794	Barley	Abscisic stress-ripening protein 2, involved in plant growth and development	[235]
BART1_0-P29183	Barley	Sucrose synthase	[235]
BART1_0-P29382	Barley	Hexosyltransferase	[235]
BART1_0-P21831	Barley	2C-type protein phosphatase protein	[235]
BART1_0-P15058	Barley	Putative zeaxanthin epoxidase	[235]
mtlD	*Escherichia coli*	Improved biomass and mannitol accumulation	[248]
DHN1	Maize	Dehydrins, help keeping cellular stability	[249]
KNR6	Maize	KERNEL NUMBER PER ROW6, which interacts with GTPase leading to increase the kernel number and to enhance the maize grain yield	[250]
YIGE1	Maize	Encodes an unidentified protein that may have a role in sucrose and auxin signaling pathways, which could have an impact on the activity of the inflorescence meristem (IM) and on the maize inflorescence morphogenesis	[251]
EAD1	Maize	Encodes a protein with cytoplasmic membrane localization that is selectively expressed in xylem conduit tissues of early maize ears and an aluminum-activated malate transporter.	[252]
AtGA2ox1	Maize	Preservation of cellular osmotic potential, regulating gibberellins content and enhancing transgenic maize's drought resistance.	[253]
LEA5-D-like	Maize		[254]
SbER2–1	Maize	Present in plasma membrane and chloroplast. The transgenic maize have enhanced photosynthesis rate	[255]

		and phenylpropanoid metabolism that promotes drought tolerance.	
qEL7	Maize	KERNEL EAR LENGTH7 increases the ethylene level, which play a role in auxin biosynthesis and boost grain yield	[256]
ZmNF-YB16	Maize	Transgenic maize showed high rate of photosynthesis and cellular antioxidant capacity during the vegetative and reproductive stage.	[257]
ZmPYL7	Maize	The expression of this gene in shoots and roots regulates the ABA level and enhances drought tolerance	[258]
ahb2	Maize	Induce fast stomata closure to reduce water lost during dehydration stress.	[259]
BADH	*Atriplex hortensis*	Higher BADH activity under normal growth	[260]
